# Immediate latissimus dorsi pedicle flap reconstruction following the removal of an eight kilogram giant phyllodes tumour of the breast: a case report

**DOI:** 10.1186/1752-1947-5-44

**Published:** 2011-01-28

**Authors:** Romesh Sarvanandan, Ramesh Thangaratnam, Ai Chen Leong

**Affiliations:** 1 Northwest Thames Deanery, 118 Sixth Avenue, London, E12 5PU, UK; 2 Hospital Sungai Buloh, Selangor, Malaysia

## Abstract

**Introduction:**

Phyllodes tumors account for less than 1% of breast tumors in women, and giant phyllodes tumors are those that are larger than 10 cm in diameter. Removal of such large tumors places a huge burden on the surgeon to reconstruct a breast that is aesthetically acceptable by the patient. We report what may be the largest giant phyllodes tumor and, most likely, the first latissimus dorsi flap used to cover such a large defect caused by the resection.

**Case presentation:**

We report the case of a 36-year-old Malaysian woman who presented with a three-year history of gradually increasing swelling of the left breast, with skin changes. Examination revealed a huge, globular, lobulated mass measuring 400 mm by 350 mm. The patient had a mastectomy with an immediate latissimus dorsi pedicled myocutaneous flap reconstruction. The breast weighed 8.27 kg, and *ex vivo*, the tumor measured 280 mm by 250 mm by 180 mm. Histopathologic analysis confirmed the diagnosis as a giant phyllodes tumor. At 12-month follow-up, the patient reports no complications and is satisfied with the aesthetic outcome.

**Conclusion:**

Giant phyllodes tumors are very rare tumors that can reach up to 40 cm in diameter. Reconstruction of such a defect is a great challenge, and we report what we believe is the first latissimus dorsi flap to cover successfully a defect of approximately 400 mm by 350 mm.

## Introduction

Phyllodes tumors are rare fibroepithelial tumors that account for about 0.4% of all female breast tumors. Clinically it resembles a fibroadenoma and can be mistakenly left alone. Giant phyllodes tumors are those larger than 10 cm and account for about 20% of all phyllodes tumors [1]. Although large tumors have been demonstrated in the past [1], a literature review did not show any previous reports of the use of latissimus dorsi flaps to cover such defects caused by the removal of tumors of comparable size to that in this case.

## Case presentation

A 36-year-old married Malaysian woman with three breast-fed children presented to our outpatient clinic with a three-year history of a gradually increasing left-sided breast mass. The mass was not associated with any pain or nipple discharge. She denied any weight loss or loss of appetite and had no history of trauma.

She had no past medical history or family history of note and had never smoked or drunk alcohol.

Before the presentation to the clinic, she went to six different traditional healers, who prescribed her topical oils, massaged her breast, and performed prayers for her. These traditional methods did not alleviate her symptoms, and finally she consulted us for further management, as she could no longer hide the huge size of her left breast.

**Figure 1:**
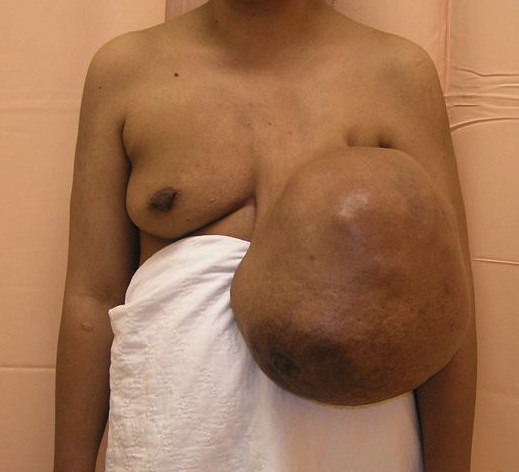
**Anterior view of the breast tumor on presentation to our clinic**.

**Figure 2:**
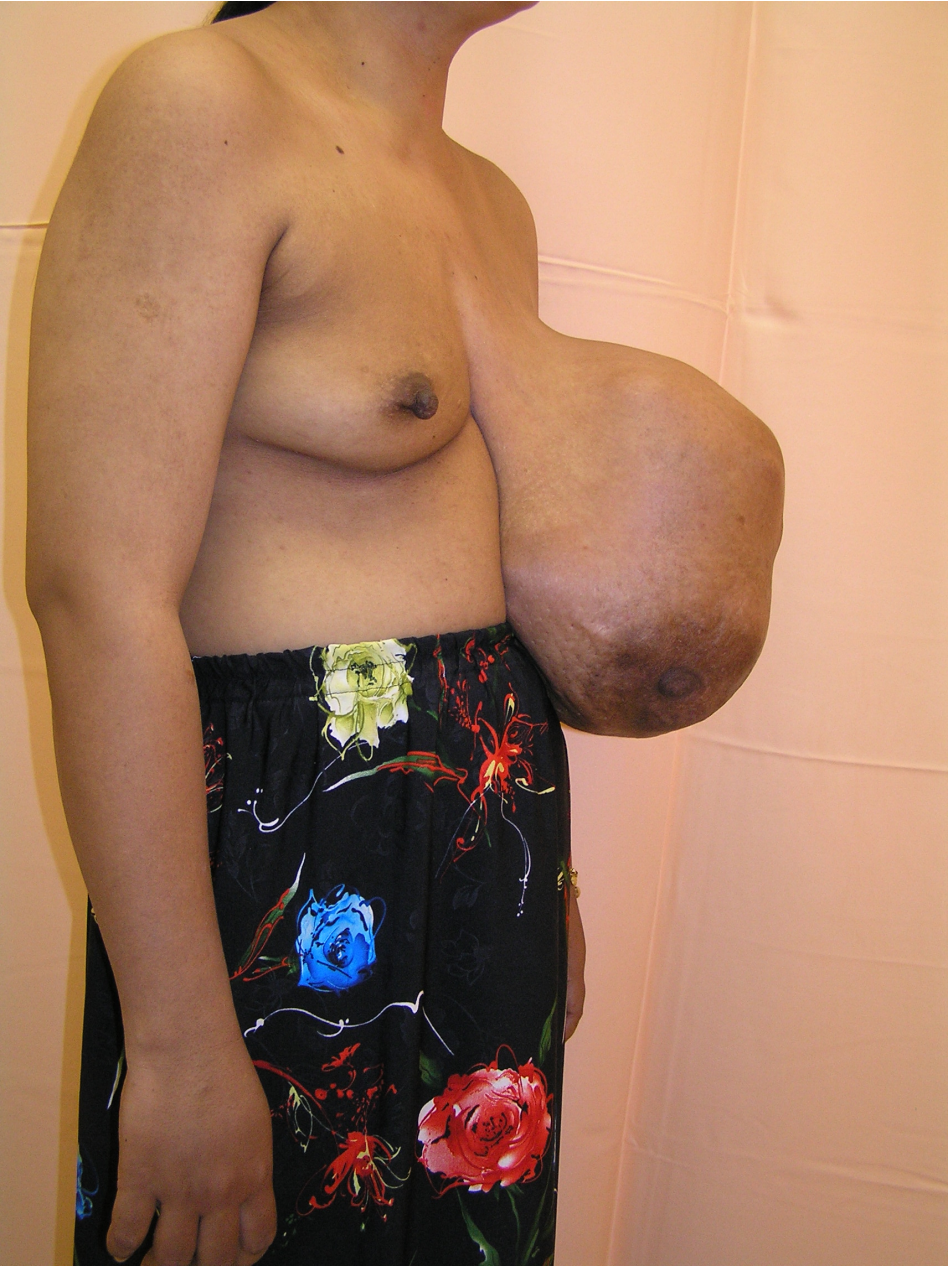
**Right lateral view of the breast tumor at presentation**.

Examination revealed a huge, globular, lobulated mass measuring 40 cm × 35 cm with an inferomedially deviated and retracted nipple with *peau d'orange *skin changes (Figures 1 and 2). A mammogram was not possible because of the size of the lesion, and a core biopsy revealed a phyllodes tumor. A computed tomography (CT) scan showed no evidence of local or distant metastasis.

**Figure 3:**
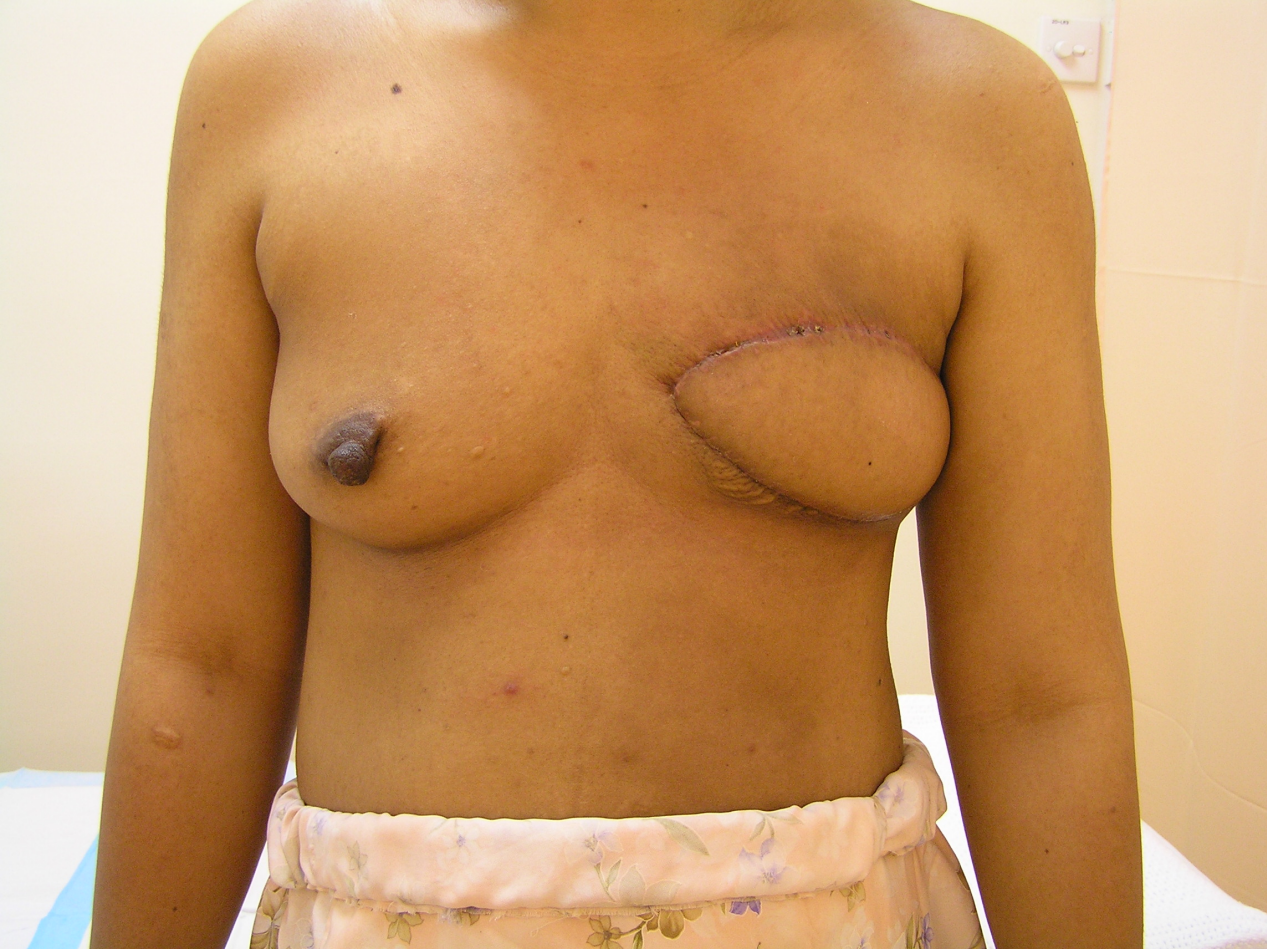
**Four months after surgery**. Anterior view of the breast four months after the mastectomy and immediate latissimus dorsi reconstruction.

The options for treatment were discussed with the patient regarding the removal of the tumor and the reconstruction, and taking in to account the size of the lesion, a mastectomy was the method of choice. As she had reduced abdominal subcutaneous tissue for a transverse rectus abdominus myocutaneous (TRAM) flap, she elected a latissimus dorsi pedicled myocutaneous flap (Figure 3). In addition to the mastectomy, our patient also underwent a level II axillary clearance because of the high suspicion of malignancy.

The resected breast weighed 8.27 kg, and the nipple-areolar complex was sacrificed in view of a possible malignant phyllodes tumor of the breast. As the patient had strong religious beliefs, she refused the offer of an expander/implant to provide additional breast volume.

**Figure 4:**
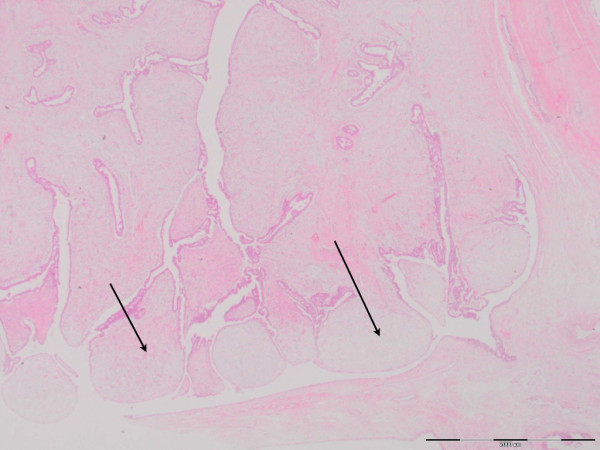
**H&E staining of the breast tissue (×1.25 magnification)**. This low-power view shows the "leaf-like" growth pattern typically seen in phyllodes tumors because of the overgrowth of stroma. Here the "leaf-like" areas project into dilated luminal space (arrows).

**Figure 5:**
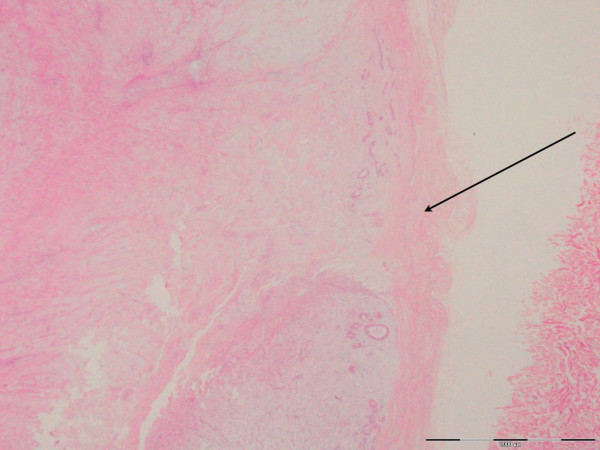
**Phyllodes tumor border H&E staining (×1.25 magnification)**. This section is a low-power view showing the border of the phyllodes tumor in this case (arrow), which is "well-circumscribed" and "pushing," rather than "invasive" (invasion would indicate malignant change). This well-circumscribed border favors a benign diagnosis.

**Figure 6:**
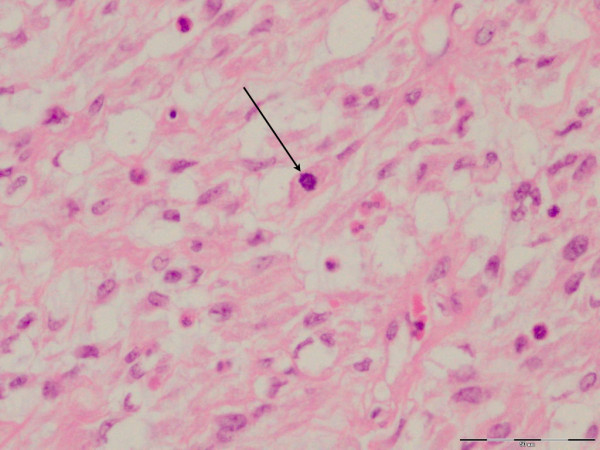
**Phyllodes tissue H&E staining (×40 magnification)**. High-power view shows the cellularity of the stroma, with presence of mitosis (arrow) and moderate cellular pleomorphism. The presence of increased mitotic activity (five per 10 high-powered fields (hpfs) in this patient), as well as the moderate cellular pleomorphism, indicates a diagnosis of "borderline" phyllodes tumor.

Microscopically, the mass was composed of compressed ducts lined by two-tiered epithelium arranged in clefts and surrounded by an overgrowth of stroma arranged in a leaf-like structure (Figures 4 and 5). A borderline giant phyllodes tumor was diagnosed after the identification of five mitoses per 10 high power fields (hpfs) at the most mitotically active area, and a predominantly expanding border with no overt malignant features (Figure 6). The tumor had a minimum of 4 cm clear surgical margin, and no removed nodes exhibited any evidence of malignancy. Our patient did not receive any adjuvant therapy (post-operative radiotherapy or chemotherapy) because of the absence of malignant features.

## Discussion

Phyllodes tumors are rare tumors of the breast that account for about 0.5% [1] of all female breast tumors. The World Health Organisation criterion is used to classify the phyllodes tumor as benign, borderline, or malignant, depending on the histologic analysis of the lesion.

Giant phyllodes tumors are those that are more than 10 cm in diameter, and account for about 20% of phyllodes tumors [1]. A review of the literature revealed two cases of giant phyllodes tumor that are of comparable size to that of our patient. The first patient had a transverse rectus abdominis myocutaneous flap (TRAM) reconstruction [2], whereas the other did not have a reconstruction as a result of direct invasion into the chest wall [1].

Although surgery is the method of treatment for phyllodes tumor of the breast, the best type of surgery for benign or borderline tumors is debatable (wide local excision or total mastectomy). However, for tumors of similar size to that of our patient, we recommend a total mastectomy to ensure complete removal of the tumor, and an immediate or delayed reconstruction of the breast to provide an added psychological benefit.

Autologous latissimus dorsi flap has long been the standard procedure for breast reconstruction until the introduction of TRAM and DIEP (deep inferior epigastric perforator) flaps. Its advantages include minimal functional morbidity as a result of harvesting the muscle, low risk of flap necrosis (1%) [3] as a result of unsevered neurovascular supply, reduced donor-site morbidity [4], in addition to its superiority in high-risk patients (diabetics, smokers, obese, and so on) who may be unsuitable for TRAM or DIEP flaps [3,4].

The commonest short-term complication is donor-site seroma collection. In general, patients younger than 50 years had a significantly lower incidence of seroma (17%), compared with those older than 50 years (33%) [5]. Long-term complications include shoulder weakness, donor-site pain, in addition to the development of capsular contracture in some patients with implants [3].

We propose that after a mastectomy for the removal of giant phyllodes tumor, a latissimus dorsi myocutaneous flap can be successfully used to provide an excellent aesthetic outcome for most patients [6].

## Conclusion

Giant phyllodes tumors are rare, and mastectomy is preferred for complete tumor excision. Latissimus dorsi myocutaneous flap can be successfully used to reconstruct the breast after the removal of very large tumors and can provide excellent aesthetic results with a low risk of flap necrosis or donor-site morbidity, and can also be used for high-risk patients who may be unsuitable for TRAM or DIEP flaps.

## Competing interests

The authors declare that they have no competing interests.

## Consent

Written informed consent in Malay (the patient does not read or speak English) was obtained from the patient for publication of this case report and accompanying images. A copy of the written consent in Malay and/or official translation can be made available for review by the Editor-in-Chief of this journal.

## Authors' contributions

RS used all the data available and wrote majority of this report. RT was the main consultant surgeon involved in the management of this patient and supplied the principles of surgical information in this article. AL saw the patient in clinic and contributed the case history notes used in this report. All authors read and approved the final manuscript.
